# Ethiopian Orthodox Fasting and Lactating Mothers: Longitudinal Study on Dietary Pattern and Nutritional Status in Rural Tigray, Ethiopia

**DOI:** 10.3390/ijerph15081767

**Published:** 2018-08-17

**Authors:** Beruk Berhanu Desalegn, Christine Lambert, Simon Riedel, Tegene Negese, Hans Konrad Biesalski

**Affiliations:** 1 College of Agriculture, Hawassa University, Postal code: 05, Hawassa, Ethiopia; tegenengss38@gmail.com; 2 Institute of Biological Chemistry and Nutrition, University of Hohenheim, Garbenstr. 30, 70593 Stuttgart, Germany; christine.lambert@uni-hohenheim.de (C.L.); simon.riedel@uni-hohenheim.de (S.R.); biesal@uni-hohenheim.de (H.K.B.)

**Keywords:** lactating mothers, Ethiopian Orthodox lent fasting, ASFs consumption, underweight, Ethiopia

## Abstract

About half of Ethiopians belong to the Orthodox Tewahedo religion. Annually, more than 200 days are dedicated to religious fasting, which includes abstaining from all types of food, animal source foods, and water. However, the association of fasting with undernutrition remains unknown in Ethiopia. Therefore, dietary pattern and nutritional status of lactating women during lent fasting and non-fasting periods were studied, and predictor variables for maternal underweight were identified. To achieve this, lactating mothers in lent fasting (*N* = 572) and non-fasting (*N* = 522) periods participated from rural Tigray, Northern Ethiopia. Average minimum diet diversity (MDD-W) was computed from two 24-h recalls, and nutritional status was assessed using body mass index (BMI). Binary logistic regression was used to identify potential predictors of maternal underweight. Wilcoxon signed-rank (WSRT) and McNemar’s tests were used for comparison of the two periods. The prevalence of underweight in fasting mothers was 50.6%. In the multivariate logistic regression model, younger age, sickness in the last four weeks preceding the survey, fasting during pregnancy, lactation periods, grandfathers’ as household decision makers, previous aid experience, non-improved water source, and not owning chicken were positively associated with maternal underweight. In WSRT, there was no significant (*p* > 0.05) difference on maternal body weight and BMI between periods. The average number of meals, diet diversity, and animal source foods (ASFs), consumption scores were significantly increased in non-fasting compared to fasting periods in both fasting and non-fasting mothers (*p* < 0.001, *p* < 0.05, and *p* < 0.001, respectively). Consumption of dark green leafy vegetables was higher in the fasting period (11%) than non-fasting (3.6%), in the study population. As a conclusion, Ethiopian Orthodox fasting negatively affected maternal nutritional status and dietary pattern in rural Tigray, Northern Ethiopia. To reduce maternal malnutrition in Ethiopia, existing multi-sectoral nutrition intervention strategies, should include religious institutions in a sustainable manner.

## 1. Introduction

Undernutrition is a serious public health problem worldwide. It is the underlying cause for 3.5 million deaths, 35% of the disease burden in children younger than 5 years old, 11% of total global disability-adjusted life-years (DALYs) and accounted for at least 20% of maternal mortality [1,2,3,4]. According to Food and Agriculture Organization of the United Nations (FAO) estimates, the global prevalence of chronically undernourished people increased from 777 million (10.6%) in 2015 to 815 Million (11%) in 2016; however, the deterioration was most severe in sub-Saharan Africa. Eastern Africa is one of the four sub-regions in sub-Saharan Africa, where one-third (33.9%) of the population was estimated to be undernourished in 2016 [3]. 

Good nutritional status of women is important for their good health and working capacity, as well as for the health of their offspring [1]. During pregnancy and lactation, women are more vulnerable to undernutrition than others at reproductive age, due to increased energy and nutrient requirements [5,6,7]. Globally in 2011, the prevalence of anemia in pregnant women was 38.2% vs. 29% in non-pregnant women [8]. According to the Demographic and Health Survey (DHS) in Ethiopia, the prevalence of anemia in pregnant or lactating women (19% and 29%) was higher than in non-lactating-non-pregnant women (15% and 21%) (DHS 2011, 2016, respectively) [9,10]. Likewise, the prevalence of underweight (BMI < 18.5 kg/m^2^) in lactating mothers was 25–55%, which was higher than non-pregnant-non-lactating women (22%) [5,9,10,11,12]. 

Food taboos refer to those foods which are strictly forbidden for health, cultural, and religious reasons [13,14,15,16]. In Ethiopia, food taboos are thought to have been established during pregnancy as a means of protecting the health of women and their babies. As a result, scholars in Ethiopia focused on the exploration of food taboos during pregnancy and its association with their nutritional status and feeding practices [15,16,17,18]. However, lactating mothers who even need more nutrients than pregnant women [5,6,12], are ignored in this regard. Religious fasting is one of the categories of dietary or food taboos, which may affect the dietary intake and nutritional status of lactating mothers, and these may lead to undernourished breastfed children. The main difference between religious fasting and the ordinary type of food taboo is its momentary nature, in which abstention from eating animal source foods and/or from eating certain foods is done for fixed periods of time [19]. According to previous studies, Ramadan fasting affected nutritional status, dietary nutrient intake, birth outcome, breast milk composition, and health status of women in reproductive age [20,21,22,23,24,25,26,27,28]. 

In Ethiopia, about half (44%) of the whole population are Ethiopian Orthodox Christians [9]. Religious fasting from any animal source foods and abstaining from any foods and water for some hours daily is mandatory. However, if it is practiced, it affects over 200 days annually [29]. Apart from this, children less than seven years old, soldiers, severely ill or weak people, pregnant, and lactating mothers are permitted to eat both animal source foods and other foods including water, without abstention during the religious fasting periods or days [19,29,30]. Nonetheless, information is lacking on the effect of religious fasting on the dietary and nutritional status of women in reproductive age. Therefore, the purpose of this study was to explore the dietary pattern and nutritional status of lactating women during Ethiopian Orthodox Lent fasting and non-fasting periods, and to identify potential predictors associated with maternal underweight in rural Tigray, Northern Ethiopia. 

## 2. Materials and Methods 

### 2.1. Study Area, Design, Participants, and Sampling 

The study was conducted in the Genta Afeshum woreda of rural Tigray, Northern Ethiopia. The woreda covers an area of 1636 km^2^ with a total population of 99,112, and almost all people (99%) are Orthodox Christians. The woreda reside at an altitude between 2045 and 3314 masl. The woreda is classified as a hotspot for food insecurity [31,32,33]. In the woreda, drought, hail storms, and livestock diseases are the major disaster risks; followed by human diseases, crop diseases, pests, and flooding. Additionally, deforestation, water pollution, and soil erosion are the major environmental problems; whereas, high dependency syndrome, poor economic conditions, land shortage, severe shortage of drinking water, and poor saving are among the major vulnerability factors at the household level [34]. 

The study had a community-based longitudinal survey design, to assess the nutritional status and dietary pattern of lactating mothers. The data was collected during the Ethiopian Orthodox lent fasting period (Fasting of Jesus Christ, 15 February 2017 to 15 April 2017) and non-fasting periods (1 May 2017 to 30 May 2017). The sample size was calculated based on the prevalence of underweight in lactating mothers in the Tigray region, using the formula for estimating single population proportion and considering a 95% of confidence interval for true prevalence, and a relative precision (d) of 5%. In lactating mothers, the prevalence of underweight (BMI < 18.5 kg/m^2^) was 25% elsewhere in Tigray [5]. The total number of lactating mothers was estimated to be 3369, which was less than 10,000; therefore, the finite source population size correction formula was used. Additionally, 10% was considered as non-responses and dropout rates. Moreover, a 1.5 design effect was used on the final calculated sample size, and the final total sample size was 575. 

Multi-stage systematic random sampling was applied to obtain representative samples for the study. At first, Genta Afeshum was randomly selected out of the three GIZ Ethiopia, Nutrition Sensitive Agriculture (NSA) project woreda’s in Tigray region. Out of twenty rural kebeles in the Genta Afeshum woreda, seven were randomly selected. Then, the list of households which had lactating mothers with children aged between 6 to 23 months old, who fulfilled the inclusion criteria, was prepared for the seven kebeles (lowest local administrative unit) by health extension workers at the nearby health posts. Subsequently, the samples were chosen using systematic random sampling techniques. 

### 2.2. Data Collection

Ten trained and well experienced data collectors who were fluent in Tigrigna, Amharic, and English languages were recruited. Additionally, before conducting the main survey, the questionnaire was translated to Tigrighna by a professional translator and verified by data collectors. Then the translated questionnaire was pre-tested for its appropriateness, by administering it to lactating mothers around Mekele, and corrections were made. Structured and semi-structured questionnaires were prepared to collect information on socio-demographic and economic characteristics, maternal and child characteristics, water, sanitation and hygiene (WASH), feeding practices, and household food security indicators [35]. 

Before conducting the study, the whole study protocol was ethically approved by the Institutional Review Board of the College of Health Sciences at Hawassa University and the Tigray Region Health Bureau in Ethiopia; and the ethical review committee of Landesärztekammer Baden-Württemberg, Germany. Permissions from Genta Afeshum Woreda Health Office were also obtained. After the purpose of the study was explained to the study participants, agreement to participate in the study was documented by signing the informed consent. Each participant was also told that the collected information was confidential, and whenever she wanted to discontinue, withdrawal from the study was possible. 

### 2.3. Minimum Women Diet Diversity Score

The minimum-diet diversity score (MDD-W) was obtained by (a) collecting two 24-h dietary recalls; (b) categorizing as consumed or not consumed of the food group, considering the minimum amount (15 g) of any food items or the sum of food items eaten under a given food group and giving a score of 1 if consumed, otherwise 0 if not; (c) calculating the diet diversity score for each two days using 10 food groups, as the summation of consumed food groups or scored 1 for each day separately; and (d) taking the average diet diversity score of the two days, as an individual MDD-W score. The 10 food groups used for calculating MDD-W score were grains, white roots and tubers, and plantains; pulses (beans, peas, and lentils); nuts and seeds; dairy; meat, poultry, and fish; eggs; dark green leafy vegetables; other vitamin A-rich fruits and vegetables; other vegetables; and other fruits [36]. 

### 2.4. Household Food Insecurity Information

Household food insecurity data was collected using the household food insecurity access scale (HFIAS). It is the measure of the degree of food insecurity (access) in the household in the past 4 weeks (30 days). The questionnaire encompassed nine questions, which assess the occurrence of food insecurity in increasing level. Under each of the nine questions, frequency of occurrence questions were a follow up to determine how often the condition (1 = rarely, 2 = sometimes, 3 = often) occurred. The Household Food Insecurity Access Prevalence (HFIAP) status indicator, was used to determine prevalence of food insecurity to report household food insecurity. Using the HFIAP indicators four categories, the households were categorized into four levels of household food insecurity (access). These were food secure, mild, moderately, and severely food insecure. After creating these four categories, the HFIAP was calculated as the number of households with a given food insecurity category divided by the total number of households with household food insecure access category, multiplied by 100 [35]. 

### 2.5. Wealth Index

Principal component analysis (PCA) was carried out to compute the wealth index. To achieve this, 17 variables: (Transport animals (horse/donkey/mule), goat and/or sheep, household owns kerosene or lamp, owns bed, chair, table, radio, electric-mitad, bicycle, mobile phone, non-mobile phone, animal drawn cart, motor bicycle, TV, electricity, windows, and separate room for animal), which could indicate the living standard of the surveyed area were included in the analysis. The first factor that explained most of the variation (86.3%) was used to group study households. Finally, the wealth tertile was performed and categorized as higher, medium, and lower [37]. 

### 2.6. Anthropometry

Weighing body mass of the mothers was conducted using a portable digital scale (Seca 770, Hanover, Germany), working with a powered battery and measured to the nearest 0.1 kg. For height measurement, a dissembling plastic height measuring board with a sliding head bar was used and measured to the nearest 0.1 cm. During weight and height measurements, the mothers were advised to remove their jackets until they had light clothes to minimize the weight due to clothes. The measurements (height and weight) were carried out using standardized equipment and procedures in duplicate and the average values were used. Additionally, the BMI of the mothers was calculated as the weight of the mothers in kilograms divided by the square of their height in meters. The BMI values of mothers were classified in three categories as underweight, normal, and overweight (<18.5, 18.5–24.99, and ≥25 kg/m^2^), respectively [38].

### 2.7. Data Management and Analysis

Before submitting the data, variable coding was conducted in SPSS version 20. Following this, the data was entered, cleaned, and analyzed. First, frequency and crosstab were conducted to determine completeness of data and to present the results in descriptive statistics (frequency and percent). Association between outcome and potential explanatory variables, was assessed using bivariate analysis with a confidence level of 95% to declare the statistical significance. Out of all the independent variables entered in bivariate logistic regression, seventy variables with *p*-values of less than 0.25 were entered for multivariable logistic regression to identify predictor variables for maternal underweight (BMI < 18.5 kg/m^2^). *p*-value < 0.05 was used to declare the variables as predictors for the outcome variable. Hosmer and Lemeshow test and C-statistics (AOC) were conducted to assess fitness of the final model. Meanwhile, multi-collinearity was checked using the variance inflation factor (VIF) and standard error with <10 and <2 as a cutoff point, respectively. Maternal nutritional status was defined as underweight (BMI < 18.5 kg/m^2^) and normal if BMI ≥ 18.5 kg/m^2^, since the interest of this study was being underweight for logistic regression. Normality of continuous data was checked using the Kolmogorov-Smirnov test. Non-normally distributed data was analyzed using the Wilcoxon signed-rank test; whereas the dichotomous data was analyzed using McNemar’s test to detect a difference between fasting and non-fasting periods, of fasting and non-fasting mothers for their dietary pattern, separately. 

## 3. Results

A total of 572 lactating mothers who were inhabitants of Genta Afeshum district of rural Tigray, Northern Ethiopia participated during the 1st round of data collection (Ethiopian Orthodox lent fasting) period, with a response rate of 99.5%. Out of those participants in the 1st survey, 522 mothers were again involved during the non-fasting period, with a response rate of 90.8%. The lost for follow-up was due to age (>23 months) of breastfeeding child, migration to other areas, and absence during the re-visit at the 2nd round survey period. 

In this study, all mothers were from the Tigray ethnic group (100%) and almost all were Ethiopian Orthodox Christians (99.7%). Majority of mothers were married (84.8%); whereas, the rest were single, widowed, and divorced mothers. More than sixty percent of mothers were thirty years old and above in the study. However, about eight out of ten mothers were married before the age of 21 years. The proportion of mothers who were illiterate and housewives were 35.1% and 79.2%, respectively. Nearly sixty percent of mothers had children of one year and above. Majority of the households were food insecure (71%), male-headed (80%), and taking aid (food, cash, in kind) (89%) before the survey period, from local government or humanitarian organizations. Likewise, 61.4% and 75% of households had family members of more than five and resided in an area more than 15 km away from the largest market in the district, respectively (Table 1). 

As shown in Table 2, 28% and 31% of mothers were fasting during the pregnancy and lactation period of the indexed child, respectively. Beside this, 65% of mothers did not change their food intake during their lactation period. Very small proportion of mothers (13.8%), had illness within four weeks preceding the fasting period. Most of the mothers (91%), used antenatal care (ANC) services four times or more during pregnancy of the indexed child. However, mothers who received post-natal care (PNC) services after delivery were 43%. (Table 2).

Mothers less than or equal to thirty years of age, were about 1.7 times more likely to be underweight than those above thirty years (Adjusted Odds Ratio (AOR) = 1.73). Conversely, mothers who had children between 13 and 18 months of age had two times higher odds of being underweight, compared to those mothers who had children between six to twelve months of age (AOR = 2.01). The odds of being underweight for mothers fasting during their pregnancy and lactation period of the indexed child, were 1.7 and 2.8 times higher than those who were not fasting in both periods (AOR = 1.75, 2.82), respectively. Additionally, mothers who had sickness within four weeks preceding the fasting period had 3.6 times higher odds of being underweight, compared to those who were healthy (AOR = 3.62). Similarly, households whose decision makers were grandfathers and had received aid were 6.0 and 2.9 times more likely to have underweight mothers, compared to households with husbands as decision makers and those which received aid (AOR = 6.02, 2.86, respectively). Households having no access to improved water and not owning chickens, were more likely to have underweight mothers compared to their counterparts (AOR = 1.57, 1.73, respectively). However, family size, marital status, family planning method used, distance from woreda market, number of ANC visits, PNC attendance, toilet presence, and wealth index had no association with maternal underweight (Table 3). 

Variables included in the adjusted model logistic regression: Mother age, Mother fasting status during pregnancy period of indexed child, Mother fasting status during lactation period of indexed child, Decision maker on main household income, Marital status, Age of indexed child, Household previous experience to aid, Family planning use status, Distance from woreda market, ANC number visits during the pregnancy period of indexed child, Mother PNC, Household consumption water source, Toilet presence in the household, Household chicken owning status, Mother sickness last one month preceding the survey, and Wealth status tertile.

A small fraction of mothers (1.4%) had height less than 145 cm, indicating stunting. Nearly one-third of mothers had body weight ≤45 kg (32.5%) and BMI < 18.5 kg/m^2^ (33.6%), in the study population. However, the prevalence was higher in fasting mothers than non-fasting mothers, both in the fasting and non-fasting period. However, the prevalence of overweight (BMI ≥ 25 kg/m^2^) was lower in fasting mothers than non-fasting in both periods, as shown in Table 4. 

Our findings demonstrated that the number of mothers who were in the fasting sub-group and had a diet diversity score (MDD-W) less than three, were slightly more in the fasting (56.7%) than non-fasting period (45.6%). However, the proportion of fasting and non-fasting mothers who scored a diet diversity greater than three, increased from the fasting period (6.7% and 8.6%, respectively) to non-fasting period (13.8%). Fasting mothers reduced consumption of coffee in the fasting period (41%), whereas non-fasting mothers (47.2%) did not. In both study periods, all mothers consumed grains, but there was no consumption of nuts and seeds. Almost all mothers ate pulses in fasting (96.7%) and non-fasting (98.1%) periods. Conversely, except two fasting mothers in fasting period, there was no consumption of other fruit groups in both periods. Furthermore, mothers who consumed vitamin A-rich fruits and vegetables preceding the survey days in both periods were less than 1%. The overall consumption of dark green, leafy vegetables was lower in the non-fasting (3.6%) than fasting period (11%). The consumption of other vegetable groups was almost the same during non-fasting (73%) and fasting (74.1%) periods. Dairy products; meat, poultry and fish; and eggs food groups were consumed by a very low number of both fasting (0%, 0% and 1.1%) and non-fasting mothers (1.4%, 0.3% and 1%) during the fasting period, respectively. However, consumption of the three ASFs significantly increased in both fasting and non-fasting mothers, in the non-fasting period compared to fasting period, as shown in Table 4. According to McNemar’s test, there was no significant difference on the proportion of fasting mothers who consumed pulses (*p* = 0.289), dark green leafy vegetables (*p* = 0.078), vitamin A-rich fruits and vegetables (*p* = 1.000), and other vegetables groups (*p* = 1.000), between fasting and non-fasting periods. However, the proportions of fasting and non-fasting mothers who consumed eggs, were significantly higher in non-fasting than fasting periods (*p* = 0.006, *p* = 0.003, respectively). Similarly, the consumption of pulses and other vegetable groups by non-fasting mothers were not significantly different (*p* = 0.332, *p* = 0.716, respectively), between fasting and non-fasting periods. However, the number of non-fasting mothers who consumed dairy; meat, poultry and fish products; eggs; and dark green leafy vegetables were significantly different (*p* = 0.002, *p* ≤ 0.001, *p* = 0.003 and *p* ≤ 0.001, respectively), between fasting and non-fasting periods (Table 5). 

Using the Wilcoxon signed-rank test, the median BMI, body weight of fasting mothers, and number of cups of coffee consumed were not significantly different between fasting and non-fasting periods. Similarly, the median BMI and body weight of non-fasting mothers were not statistically different between the two periods (Table 6 and Table 7). However, in both fasting and non-fasting mothers, the number of meals eaten (*p* ≤ 0.001), MDD-W (*p* = 0.037 and *p* = 0.014), and ASFs consumption scores (*p* ≤ 0.001) were significantly increased in the non-fasting than fasting periods, respectively. However, the number of cups of coffee consumed by non-fasting mothers was significantly (*p* = 0.009) higher in fasting period than in the non-fasting period (Table 6 and Table 7).

## 4. Discussion

This study assessed whether nutritional status and dietary pattern of lactating mothers were different between Ethiopian Orthodox lent fasting and non-fasting periods. It also determined factors associated with maternal underweight in rural Tigray, Northern Ethiopia. 

### 4.1. Nutritional Status of Lactating Mothers 

Adult stature is the collective outcome of the interaction between environment and inheritances, over the critical growing period of a person [39]. Prior evidence has demonstrated that short maternal height was associated with increased offspring mortality, underweight, and stunting in infancy, childhood, and later in an adult age [39,40]. In our study, prevalence of maternal stunting was 1.4%, which was less than previous findings in Ethiopia [5,9,10,41]. Shorter women are believed to have reduced protein and energy stores, smaller size of their reproductive organs, and smaller pelvis diameter. This may limit fetal development in the uterus, increases risk for mother and child complication during delivery, and later infant growth through reduced breast milk quantity and quality, resulting in stunted children. Thus, appropriate feeding behavior is important for pregnant women health, and later for bearing healthier and well grown new born babies. This finding suggests that health extension agents working in the rural communities should advise mothers on appropriate feeding behavior during the pregnancy period. 

The overall prevalence of maternal underweight in this study was between 32.6 and 33.6%. The prevalence was lower than previous findings in Tigray region (34–55%), and Dedo and Seqa-Chekorsa districts (41%), in South-west Ethiopia [9,10,11,12]. Conversely, the prevalence was higher than in other studies in the Tigray and Oromia regions of Ethiopia [5,41,42,43,44]. The latter could be related to climate phenomenon ‘El Nino’, which caused the strongest famine in Ethiopia, where the impact seriously affected Tigray region. It could also be related to differences in feeding practices, study population, and period [45].

Shockingly, prevalence of underweight was 51% in fasting mothers compared with non-fasting mothers (25%). The result was consistent with studies conducted on lactating mothers living in the midland agro-ecology of Tigray region, which was 57% [12]. Similarly, the BMI was significantly lower among fasting adults than non-fasting adults in Greek Orthodox Christians [28]. This could be related to the almost 317 kcal difference between fasting and non-fasting adults, in end-holy days of fasting periods [46]. 

In the present study, prevalence of overweight was between 1.1% and 3.8%, respectively, which was relatively comparable with the Ethiopian Demographic and Health Survey (EDHS) 2011 report (2.9%) in Tigray, and elsewhere (1.3–1.8%) in the region [5,24,38], but lower than the EDHS (2016) report at national level (6%) and Tigray region (4.9%). In urban women, it was reported (6.2–25.3%) in Ethiopia, Bengal district in India (5.4%), and Nepal (6.3–24.8%) [44,47,48,49,50,51,52]. This might be because we attributed this finding to rural people [10], where most could engage in heavy physical activities and walking over long distances to access services due to the mountainous topography [53,54]. 

Mothers thirty years of age and younger were 1.7 times more likely to be underweight than those above thirty. That conforms with other studies conducted in Ethiopia and Nepal, resulting in higher prevalence of underweight in younger women [10,47]. In the current study, more than 27% of mothers fasted during their pregnancy period, and these mothers had 1.7 times more odds to be underweight than not fasting during the same period. In a previous study, more than one-third of mothers were fasting during their pregnancy period in Oromia region, Ethiopia [15]. Similarly, those mothers fasting during lactation period were 2.9 times more exposed to underweight, than those who did not practice.

Mothers who had children between the age of 13 and 18 months, were twice more likely to be underweight compared to those who had a child aged 6–12 months. Similar result was observed in a study conducted by Haileslassie and his colleagues in Northern Ethiopia [5]. This might be due to increased nutritional requirements of the growing child, effort for child care in connection with food intake by the mother that is not increased or even decreased. 

Disease is one of the immediate causes of maternal and child undernutrition [1]. Mothers who had any illness in the last four weeks preceding the survey were 3.6 times more frequently underweight than healthy mothers. In a study conducted in the Limu area of Southern Ethiopia, maternal sickness was positively associated with maternal underweight [55]. This could be related to decreased food intake and absorption, alteration of metabolism, and increment in nutritional requirements. Marital status of lactating mothers was not significantly associated with maternal underweight. Prior evidence has also demonstrated that marital status was not associated with Ethiopian women [56]. Otherwise, birth spacing has important implications for the health and nutritional status of mothers and their children [57]. In our study, family planning use was not associated with maternal underweight, which is inconsistent with the study conducted in the Tena district of Oromia region, Ethiopia. In the latter, the prevalence of family planning utilization was higher (65%) than our finding, which was 52% [58]. 

Good care during pregnancy is important for the health of the mother and development of the unborn baby. The findings of this study indicated that a major proportion (91%) of mothers had at least four ANC services during their pregnancy period, and were not significantly associated with maternal underweight. This result was equal with the regional coverage of Tigray (91%), but higher than the national prevalence (62%) [10]. The postnatal period is a critical phase in the lives of a mother and the newborn baby. In this period, major changes occur, but it is the most neglected time for the provision of quality services. As a result, the rates of provision of skilled care are lower after childbirth when compared to rates before and during childbirth [59]. In this study, prevalence of PNC coverage was 42.5%, which is comparable with previous findings in Tigray region (45.4%); however, PNC attendance was not significantly associated with maternal undernutrition. 

Household size was not significantly associated with maternal underweight in the study. This coincides with studies in Southern Ethiopia and Tigray region [5,41,55]. However, it was inconsistent with one study conducted in Nekemte town, Oromia region in Ethiopia [44]. The difference might be a higher proportion of lactating mothers who lived in rural households with many family members, in our case. Grandfathers as a decision maker for the household were associated with maternal underweight. This could be related to sharing the household income to more family members or to the loss (death, departure) of the husband resulting in lower working capacity and income, or due to most lactating mothers in grandfather headed households being younger, which is associated with maternal underweight in our case. It has also been reported that mothers who had more children, decreases the resources allocated including food, resulting in underweight [60]. 

Access to safe drinking water, sanitation and hygiene (WASH) services is a fundamental element of healthy communities and has an important positive impact on child and maternal nutrition [61,62]. One-third of households included in this study, had non-improved water sources for household consumption. The odds of being underweight for mothers from non-improved water sources were 1.6 higher than those from households with improved water sources. This may be due to the fact of frequent illness related to water borne diseases and contamination. 

Accordingly, child undernutrition was associated with source of drinking water in Iraq and sub-Saharan Africa [63,64]. In contrast, toilet presence in the household was not associated with maternal underweight. This might apply to the majority of households included in the study, one of the successes of the health extension program in Ethiopia. 

In our study, households not owning chickens were 1.8 times more likely to have underweight mothers, than those who owned chickens. Similarly, the proportion of mothers who ate more diversified foods were higher in households which owned chicken, than those from households not owning chicken. Prior research in Ethiopia, indicated that owning livestock in the household was associated with a higher diet diversity score [65]. Studies in three East African countries also evidenced that, in households owning livestock, the prevalence of child stunting was low [66]. Thus, promoting chicken husbandry may also improve the low consumption of animal source foods, and the diversity of food to be eaten at large. Among households involved in the study, more than a quarter (32.5%) were at the lowest wealth tertile. According to the EDHS report, a lower proportion of households (23%) was at the lowest wealth quantile [10]. Of thirty-four woredas in Tigray region, thirty-one were food insecure, including our study district [33]. The results from the present study showed that more than two-thirds (71%) of households were food insecure. Comparably, the prevalence of food insecurity was 76% in East Bedawacho district of Southern Ethiopia [67]. However, the prevalence was lower than in a study conducted in two agro-climatic zones in Sidama, Southern Ethiopia, which was 82% [68].

### 4.2. Dietary Patterns of Lactating Mothers

According to the essential nutrition action (ENA), mothers are recommended to take at least two additional meals during their lactation period [69]. In the present study, nearly two-thirds of the mothers (65.4%) did not change the food intake during their lactation period. This result is lower than a study conducted in Samre district which reported (71%), but higher than findings in Raya area (59%) of Tigray region. This could be related to the interval in the study periods and study area [5,41]. Lactating mothers who ate more than three times a day were 9–12%. This result agreed with findings in the Tigray, Oromia, and Southern regions in Ethiopia [11,41]. One-third of lactating mothers ate less than three times on average of the two days preceding the survey during fasting period, which is lower than the expected three meals to have in a day of a normal adult in real context. Prior research in Samre district of Northern Ethiopia, showed that the proportion of lactating mothers who had less than three meals in the last 24-h preceding their survey was 27% [5], which is comparable with our findings. However, this prevalence reduced to 8.4% in the non-fasting period, which is comparable with the prevalence (7.5%) of pregnant women in Gambela town, Western Ethiopia [70]. Similarly, the number of meals eaten both by fasting and non-fasting mothers were significantly increased after two months of fasting period. The latter should be further studied for explanation.

Diet diversity is one important dimension of diet quality, and a proxy indicator for higher micronutrient adequacy [36]. Majority of mothers had a diet diversity score of three and less in the fasting period (92%) and non-fasting period (87%). This could indicate that dietary micronutrient inadequacy is high in the study population. The median diet diversity score for both sub-groups of fasting and non-fasting mothers, increased significantly in non-fasting period. This was associated with higher consumption of ASFs and a higher frequency of meals in our and related studies [65,71,72]. Thus, in the Ethiopian Orthodox fasting period, the feeding practice of lactating mothers is sub-optimal. This finding suggests that nutrition education, which will improve feeding practices should involve religious leaders in a sustainable manner. The number of mothers who had consumed more cups of coffee was higher in the fasting than non-fasting period, in the whole study population. Likewise, non-fasting mothers took significantly more coffee in the fasting period than non-fasting. However, consuming more coffee during the fasting period, where plant-based foods are the sole source of minerals, could reduce their bioavailability, leading to increased risk for micronutrient deficiencies, especially of iron, calcium, and zinc [73]. However, many fasting mothers consumed more cups of coffee in the non-fasting than fasting period. This could be referred to some Ethiopian Orthodox religion monarchist, who preached to followers not to consume coffee during fasting periods. 

In this study, cereals, pulses, and other vegetables were the main food groups commonly consumed by lactating mothers. This result agrees with previous studies in Ethiopia [65,71,74]. These plant foods usually contain dietary components that compromise digestion and inhibit absorption of vital nutrients. For example, phytic acid chelates multivalent ions such as zinc, calcium, and iron; therefore, their bioavailability reduced [75]. This finding suggests that traditional processing techniques which can improve the bioavailability of minerals should be promoted in the community. Dark green, leafy vegetables are important plant sources of micronutrients like iron, calcium, and vitamin A [76]. However, their consumption was very low in the non-fasting period, both in fasting (5.6%) and non-fasting (2.8%) mothers. However, the result was lower than that previously reported for Axum town (19%) in Northern Ethiopia. The discrepancy between these two findings could be related to better market access, which is less in this study due to the rural area. However, the number of non-fasting mothers who consumed dark green, leafy vegetables was significantly higher in the fasting period than non-fasting period. This could be related to a potential to choose and consume more diversified food, especially ASFs which were less consumed in fasting period. Furthermore, almost all lactating mothers did not consume nuts and seeds, fruits, and vitamin A rich fruits and vegetables food groups at all in both study periods, because of a lack of cultivation on local farms and unavailability in the market [77]. Thus, activities which can improve the dietary diversity including nuts and seeds, dark green leafy vegetables, and vitamin A and C rich fruits consumption, as well as meal frequency are inevitable. Moreover, the consumption of fruits which are rich in vitamin C should be taught to improve the mineral bioavailability of plant foods, which are the predominate source of most nutrients for a given community. 

The proportion of lactating mothers who consumed ASFs was significantly lower in the fasting than non-fasting period. In Northern Ethiopia, particularly in rural Tigray, most strict Ethiopian Orthodox Christians abstain from eating animal source foods during the lent and other fasting periods or days, including Wednesday and Friday. During these fasting periods, the demand for cattle meat was observed to be low, resulting in closure of abattoirs or minimizing the service provided [78]. A previous study, reported that more than 85% of butcher houses were closed in Addis Ababa during Wednesday and Friday, which are Orthodox Christians fasting days of the week [79].

This study has much strength and some limitations. It is the first study in Ethiopia, which assessed the effect of religious fasting on maternal nutrition, particularly lactating mothers, who need more nutrients than pregnant women. Moreover, the study was done in an area, where almost all people were Ethiopian Orthodox Christians to minimize research bias. In addition, the study had a longitudinal nature, which considered a large sample size, and two 24-h dietary recall data for each study period. On top of these, this study also determined the differences in dietary and nutritional status during fasting and non-fasting periods, for both fasting and non-fasting mothers’ sub-groups, separately. However, the study only considered the long Ethiopian Orthodox lent fasting period out of the seven official fasting periods. This study did not measure micronutrient levels or bio-markers in the blood, to assess micronutrient deficiencies reflecting the consequences of qualitative malnutrition. Underweight only indicates energetic malnutrition, representing the tip of the iceberg.

## 5. Conclusions

It has been found that the prevalence of underweight among fasting, lactating mothers during Ethiopian Orthodox fasting periods is high and a serious public health problem in the district. The dietary pattern (diet diversity and number of meals eaten) of lactating mothers is sub-optimal, both in fasting and non-fasting periods. Cereals and pulses are the dominant food groups eaten in the district, known to have a high amount of anti-nutrients like phytic acid, which limit the bioavailability of minerals for absorption. Therefore, activities which will improve dietary diversity including nuts and seeds, dark green leafy vegetables, and vitamin A and C rich fruits consumption, as well as meal frequency are urgently needed. Additionally, consuming vitamin C rich fruits and use of traditional processing techniques, which can improve the bioavailability of minerals should be promoted. Furthermore, promoting chicken husbandry may also improve the low consumption of animal source foods. Though moderate intake of coffee is beneficial, consuming coffee closely to meals to be eaten will decrease the absorption of non-heme iron from plant source foods and should be discouraged. Thus, households with younger mothers and non-improved water sources should get priority in nutritional intervention. Generally, multi-sectoral nutrition intervention strategies should include religious institutions to reduce maternal malnutrition in Ethiopia. Otherwise, nutrition education activities will not lead to sustainable improvements.

## Figures and Tables

**Table 1:** Socio-demographic and economic characteristics of the study participants (*n* = 572) in rural Tigray, Northern Ethiopia (February–June, 2017).

Characteristics (*n* = 572)		Number	Percent
Ethnicity of mother	Tigray	572	100
Religion of mother	Orthodox	570	99.7
	Other	2	0.3
Current maternal age (years)	>30	227	39.7
	≤30	345	60.3
Mother age at marriage(years)	>20	116	20.3
	≤20	456	79.7
Mother age at giving 1st birth (years)	>20	279	48.8
	≤20	293	51.2
Family size	≤5	221	38.6
	>5	351	61.4
Household previous aid experience	No	64	11.2
	Yes	508	88.8
Household main income decision maker	Husband	129	22.6
	Jointly husband and wife	352	61.5
	Wife	74	12.9
	Grand father	17	3.0
Age of child (months)	≤12	241	42.1
	13–18	200	35.0
	>18	131	22.9
Mother previous credit experience from local institutes	No	438	76.6
	Yes	134	23.4
Distance from woreda market (km)	≤15	143	25.0
	>15	429	75.0
Marital status	Married	485	84.8
	Others	87	15.2
Mother education	Illiterate	201	35.1
	Literate	371	64.9
Mother occupation	Housewives	453	79.2
	Farmer	81	14.2
	Daily laborer	15	2.6
	Business owner	15	2.6
	Employee	8	1.4
Household head	Father	458	80.1
	Mother	114	19.9
Household food security status	Food secured	166	29.0
	Mildly food insecure	181	31.6
	Moderately food insecure	213	37.3
	Severely food insecure	12	2.1
Wealth tertile	Higher	149	26.1
	Medium	237	41.4
	Lower	186	32.5
Household chicken owning	No	247	43.2
	Yes	325	56.8

**Table 2:** Maternal health and feeding practices (*n* = 572) in rural Tigray, Northern Ethiopia (February–June 2017).

Characteristics (*n* = 572)		Numbers	Percent
Fasting during lactation	No fasting	394	68.9
	Fasting	178	31.1
Fasting during pregnancy	No fasting	414	72.4
	Fasting	158	27.6
Change of food intake	No	374	65.4
	Yes	198	34.6
Number of visits in antenatal care	≥4	518	90.6
	<4	54	9.4
Attendance of postnatal care service after delivery	No	329	57.5
	Yes	243	42.5
Household water source	Improved	379	66.3
	Non-improved	193	33.7
Household toilet presence	No	112	19.6
	Yes	460	80.4
Household garbage pit presence	No	74	12.9
	Yes	498	87.1
Family planning methods	No	274	47.9
	Yes	298	52.1
Sickness last 4 weeks	No	493	86.2
	Yes	79	13.8

**Table 3:** Association of some socio-demographic, health, and feeding practice variables with maternal underweight (BMI) during Ethiopian Orthodox Lent fasting period in Rural Tigray, Northern Ethiopia (*n* = 572).

Variables	Underweight		COR (95% CI)	AOR (95% CI)
	No	Yes		
	Number (%)	Number (%)		
Mother age				
>30	158 (69.6)	69 (30.4)	1	1
≤30	222 (64.3)	123 (35.7)	1.27 (1.11, 2.71)	1.73 (1.11, 2.71) *
Mother fasting status during pregnancy period of indexed child				
No	301 (72.7)	113 (27.3)	1	1
Yes	79 (50.0)	79 (50.0)	2.66(1.82, 3.89) *	1.75 (1.11, 2.75) *
Mother fasting status during lactation period of indexed child				
No	292 (74.1)	102 (25.9)	1	1
Yes	88 (49.4)	90 (50.6)	2.93 (2.02, 4.24) *	2.82 (1.80, 4.42) *
Family size				
≤5	159 (71.9)	62 (28.1)	1	1
>5	221 (63.0)	130 (37.0)	1.51 (1.05, 2.17)	1.42 (0.87, 2.32)
Decision maker on main household income				
Husband	83 (64.3)	46 (35.7)	1	1
Jointly husband and wife	246 (69.9)	106 (30.1)	0.78 (0.51, 1.19)	0.65 (0.39, 1.09)
Wife	46 (62.2)	28 (37.8)	1.10 (0.61, 1.98)	0.94 (0.35, 2.47)
Grand father	5 (29.4)	12 (70.6)	4.33 (1.44,13.06) *	6.02 (1.47, 24.80) *
Marital status				
Married	330 (68.0)	155 (32.0)	1	1
Others	50 (57.5)	37 (42.5)	1.58 (0.99, 2.51)	1.28 (0.52, 3.17)
Age of indexed child				
≤12 months	171 (71.0)	70 (29.0)	1	1
13–18	120 (60.0)	80 (40.0)	1.63 (1.10, 2.42) *	2.01 (1.27, 3.18) *
>18	89 (67.9)	42 (32.1)	1.15 (0.73, 1.83)	1.14 (0.67, 1.96)
Household previous experience to aid				
No	53 (82.8)	11 (17.2)	1	1
Yes	327 (64.4)	181 (35.6)	2.67 (1.36, 5.23) *	2.86 (1.32, 6.20) *
Family planning use status				
Yes	205 (68.8)	93 (31.2)	1	1
No	175 (63.9)	99 (36.1)	1.25 (0.88, 1.76)	1.12 (0.74, 1.69)
Distance from woreda market				
≤15	101 (70.6)	42 (29.4)	1	1
>15	279 (65.0)	150 (35.0)	1.29 (0.86, 1.95)	1.26 (0.72, 2.21)
ANC number visited during the pregnancy period of indexed child				
≥4	349 (67.4)	169 (32.6)	1	1
<4	31 (57.4)	23 (42.6)	1.53 (0.87, 2.71)	0.97 (0.49, 1.94)
Mother PNC				
Yes	169 (69.5)	74 (30.5)	1	1
No	211 (64.1)	118 (35.9)	1.28 (.90, 1.82)	1.01 (0.66, 1.55)
Household consumption water source				
Improved	263 (69.4)	116 (30.6)	1	1
Non-improved	117 (60.6)	76 (39.4)	1.47 (1.03, 2.12) *	1.57 (1.02, 2.43) *
Toilet presence in the household				
Yes	311 (67.6)	149 (32.4)	1	1
No	69 (61.6)	43 (38.4)	1.30 (0.85, 1.99)	1.48 (0.90, 2.43)
Household chicken owning status				
Yes	229 (70.5)	96 (29.5)	1	1
No	151 (61.1)	96 (38.9)	1.52 (1.07, 2.15) *	1.73 (1.15, 2.61) *
Mother sickness last one month preceding the survey				
No	350 (71.0)	143 (29.0)	1	1
Yes	30 (38.0)	49 (62.0)	3.99 (2.44, 6.55) *	3.62 (2.00, 6.54) **
Wealth status tertile				
Higher	134 (72.0)	52 (28.0)	1	1
Medium	153 (64.6)	84 (35.4)	0.64 (0.41, 1.02)	0.98 (0.53, 1.83)
Lower	93 (62.4)	56 (37.6)	0.91 (0.60, 1.40)	0.86 (0.52,1.41)

COR = Crude Odds Ratio; AOR = Adjusted Odds Ratio; CI = confidence interval; Hosmer and Lemeshow test showed *p* = 0.197; C-statistic: Area under the Curve (AUC) = 0.766; 95% CI (0.724–0.809); * = significantly associated at *p* < 0.05.

**Table 4:** Food consumption and anthropometric status of lactating mothers during Ethiopian Orthodox lent fasting and non-fasting periods at rural Tigray, Northern Ethiopia.

Variables		Lent Fasting Period (*n* = 572)			Non-Fasting Period (*n* = 522)		
		Non-Fasting Mothers	Fasting Mothers	Total	Non-Fasting Mothers	Fasting Mothers	Total
		*n* (%)	*n* (%)	*n* (%)	*n* (%)	*n* (%)	*n* (%)
Height (cm)	<145	7 (1.8)	1 (0.6)	8 (1.4)			
	≥145	387 (98.2)	177 (99.4)	564 (98.6)			
Weight (kg)	≤45 kg	106 (26.9)	80 (44.9)	186 (32.5)	97 (26.8)	71 (44.4)	168 (32.2)
	>45 kg	288 (73.1)	98 (55.1)	386 (67.5)	265 (73.2)	89 (55.6)	354 (67.8)
BMI (kg/m^2^)	<18.5	102 (25.9)	90 (50.6)	192 (33.6)	90 (24.9)	81 (50.6)	171 (32.8)
	18.5–24.99	277 (70.3)	86 (48.3)	363 (63.4)	259 (71.5)	77 (48.1)	336 (64.3)
	≥25	15 (3.8)	2 (1.1)	17 (3.0)	13 (3.6)	2 (1.2)	15 (2.9)
Diet diversity score (MDD-W)	<3	199 (50.5)	101 (56.7)	300 (52.5)	163 (45.0)	73 (45.6)	236 (45.2)
	3	161 (40.9)	65 (36.5)	226 (39.5)	149 (41.2)	65 (40.6)	214 (41.0)
	>3	34 (8.6)	12 (6.7)	46 (8.0)	50 (13.8)	22 (13.8)	72 (13.8)
Number of meals	<3	119 (30.2)	64 (36.0)	183 (32.0)	29 (8.0)	15 (9.4)	44 (8.4)
	3	226 (57.4)	92 (51.7)	318 (55.6)	299 (82.6)	131 (81.9)	430 (82.4)
	>3	49 (12.4)	22 (12.4)	71 (12.4)	34 (9.4)	14 (8.8)	48 (9.2)
Number of cups of coffee	<2	208 (52.8)	105 (59.0)	313 (54.7)	215 (59.4)	94 (58.8)	309 (59.2)
	≥2	186 (47.2)	73 (41.0)	259 (45.3)	147 (40.6)	66 (41.2)	213 (40.8)
Grain consumption		394 (100.0)	178 (100.0)	572 (100.0)	362 (100.0)	160 (100.0)	522 (100.0)
Pulse consumption		383 (97.2)	170 (95.5)	553 (96.7)	355 (98.1)	157 (98.1)	512 (98.1)
Nuts and seeds consumption		0 (0.0)	0 (0.0)	0 (0.0)	0 (0.0)	0 (0.0)	0 (0.0)
Dairy products consumption		8 (2.0)	0 (0.0)	8 (1.4)	23 (6.4)	9 (5.6)	32 (6.1)
Meat, poultry and fish consumption		2 (0.5)	0 (0.0)	2 (0.3)	60 (16.6)	19 (11.9)	79 (15.1)
Eggs consumption		4 (1.0)	2 (1.1)	6 (1.0)	19 (5.2)	12 (7.5)	31 (5.9)
Dark green leafy vegetables consumption		45 (11.4)	18 (10.1)	63 (11.0)	10 (2.8)	9 (5.6)	19 (3.6)
Vitamin A-rich fruits and vegetables consumption		4 (1.0)	1 (0.6)	5 (0.9)	0 (0.0)	1 (0.6)	1 (0.2)
Other vegetables consumption		294 (74.6)	130 (73.0)	424 (74.1)	265 (73.2)	116 (72.5)	381 (73.0)
Other fruits consumption		0 (0.0)	2 (1.1)	2 (0.3)	0 (0.0)	0 (0.0)	0 (0.0)

**Table 5:** Food consumption and anthropometric status of fasting and non-fasting mothers, during Ethiopian Orthodox lent fasting and non-fasting periods in rural Tigray, Northern Ethiopia.

Parameters	Fasting Mothers (a, *n* = 160)			Non-Fasting Mothers (b, *n* = 362)		
	Lent Fasting Period	Non-Fasting Period	Significance	Lent Fasting Period	Non-Fasting Period	Significance
	*n* (%)	*n* (%)	*p*-value	*n* (%)	*n* (%)	*p*-value
Grain consumption	160 (100.0)	160 (100.0)	NA	362 (100.0)	362 (100.0)	NA
Pulse consumption	153 (95.6)	157 (98.1)	0.289	351 (97.0)	356 (98.3)	0.332
Nuts and seeds consumption	0 (0.0)	0 (0.0)	NA	0 (0.0)	0 (0.0)	NA
Dairy products consumption	0 (0.0)	9 (5.1)	NA	7 (1.9)	23 (6.4)	0.002 *
Meat, poultry and fish consumption	0 (0.0)	19 (11.9)	NA	2 (0.6)	60 (16.6)	≤0.001 *
Eggs consumption	2 (1.2)	12 (7.5)	0.006 *	4 (1.1)	19 (5.2)	0.003 *
Dark green leafy vegetables consumption	18 (11.2)	9 (5.6)	0.078	41 (11.3)	10 (2.8)	≤0.001 *
vitamin A-rich fruits and vegetables consumption	1 (0.6)	1 (0.6)	1.000	4 (1.1)	0 (0.0)	NA
Other vegetables consumption	116 (72.5)	116 (72.5)	1.000	270 (74.6)	265 (73.2)	0.716
Other fruits consumption	1 (0.6)	0 (0.0)	NA	0 (0.0)	0 (0.0)	NA

Data analysis using McNemar’s test, significant level at *p* < 0.05, a < b, NA-the data was not appropriate for analysis; * = significantly associated at *p* < 0.05.

**Table 6:** Comparison of fasting mothers’ food consumption and anthropometric status between Orthodox lent fasting and non-fasting periods in rural Tigray, Northern Ethiopia.

Variables (*N* = 160)	Data Collection Period						
	Lent Fasting (a)			Non-Fasting (b)			
	Mean (SD)	Median (IQR)	Range (Min, Max)	Mean (SD)	Median (IQR)	Range (Max, Min)	Sign
Weight (kg)	46.18 (5.55)	45.55 (42.23, 49.48)a	(35.40, 65.60)	45.98 (5.49)	45.65 (42.23, 48.90)a	35.30, 65.40	0.054
BMI (kg/m^2^)	18.86 (2.14)	18.47 (17.55, 20.05)a	(14.79, 27.99)	18.79 (2.18)	18.48 (17.49, 20.0)a	14.75, 28.21	0.051
Diet Diversity Score (MDD-W)	2.57 (0.54)	2.50 (2.0, 3.0)a	(1.00, 4.0)	2.69 (0.58)	3.0 (2.0, 3.0)b	1.50, 4.0	0.037
ASFs Score	0.01 (0.06)	0 (0, 0)a	(0, 0.50)	0.13 (0.27)	0 (0, 0)b	0, 1.0	≤0.001
Number of meals	2.79 (0.49)	3.0 (2.5, 3.0)a	(1.50, 4.0)	3 (0.29)	3.0 (3.0, 3.0)b	2.0, 4.50	≤0.001
Number of cup of coffee	2.64 (3.4)	1.5 (0, 3.0)a	(0, 12.0)	1.74 (1.67)	1.5 (0, 3.0)a	0, 7.50	0.058

Data analysis using Wilcoxon Signed Ranks Test significant level at *p* < 0.05, a < b.

**Table 7:** Comparison of non-fasting mothers’ food consumption and anthropometric status between Orthodox Lent fasting and non-fasting periods in rural Tigray, Northern Ethiopia.

Variables (*N* = 362)	Data Collection Period						
	Lent Fasting (a)			Non-Fasting (b)			
	Mean (SD)	Median (IQR)	Range (Min, Max)	Mean (SD)	Median (IQR)	Range (Max, Min)	Sign
Weight (kg)	49.30 (6.41)	48.50 (45.10, 53.10)a	35.50, 72.00	49.20 (6.61)	48.50 (44.60, 53.13)a	35.70, 74.00	0.092
BMI (kg/m^2^)	20.08 (2.36)	19.68 (18.53, 21.34)a	15.87, 29.91	20.04 (2.43)	19.64 (18.50, 21.48)a	15.57, 29.79	0.086
Diet Diversity Score (MDD-W)	2.63 (0.53)	2.50 (2.00, 3.00)a	1.00, 4.00	2.73 (0.55)	3.00 (2.00, 3.00)b	1.50, 4.50	0.014
ASFs Score	0.02 (0.12)	0.00 (0.00, 0.00)a	0.00, 1.00	0.16 (0.31)	0.00 (0.00, 0.00)b	0.00, 1.50	≤0.001
Number of meals	2.84 (0.50)	3.00 (2.50, 3.00)a	1.00, 4.50	3.01 (0.27)	3.00 (3.00, 3.00)b	2.00, 4.50	≤0.001
Number of cup of coffee	2.12 (1.96)	1.5 (0.00, 3.00)b	0.00, 9.00	1.76 (1.57)	1.5 (0.00, 3.00)a	0.00, 6.00	0.009

Data analysis using Wilcoxon Signed Ranks Test significant level at *p* < 0.05, a < b.
